# Characterization of the depolarization contrast line in the outer retina using polarization-sensitive optical coherence tomography

**DOI:** 10.1038/s41598-025-11111-w

**Published:** 2025-07-14

**Authors:** Saori Igarashi, Michiko Mandai, Kota Totani, Masahiro Yamanari, Daiki Sakai, Midori Yamamoto, Yasuhiko Hirami, Satoshi Sugiyama, Yasuo Kurimoto

**Affiliations:** 1 Department of Ophthalmology, Kobe City Eye Hospital, 2-1-8 Minatojima Minamimachi, Chuo-ku, Kobe-shi, 650-0047 Hyogo Japan; 2 Department of Ophthalmology, Kobe City Medical Center General Hospital, 2-1-1 Minatojima Minamimachi, Chuo-ku, Kobe-shi, 650-0047 Hyogo Japan; 3 Tomey Corporation, 2-11-33 Noritakeshinmachi, Nishi-ku, Nagoya-shi, 451-0051 Aichi Japan

**Keywords:** Optical coherence tomography, Retina, Fovea, Medical research, Optical imaging, Biological techniques, Imaging

## Abstract

This study aimed to characterize the anatomical features of a new observation, the depolarization contrast line (DCL) near the photoreceptor inner/outer segment (IS/OS) area visualized using polarization-sensitive optical coherence tomography (PS-OCT). Twenty-eight participants (56 eyes) with no fundus disease were included. Horizontal and vertical B-scan images, averaged from 100 frames passing through the fovea, were analyzed using polarimetric entropy, a dimensionless parameter reflecting depolarization. The mean entropy at the DCL was 0.191 ± 0.043, with a correlation between horizontal and vertical directions (R² = 0.577, *p* < 0.0001) and between the right and left eyes (R² = 0.240, *p* = 0.046). In 25 eyes with good image quality, entropy showed a negative correlation with age (R² = 0.217, *p* = 0.019). In 11 eyes analyzed with a distinct DCL, this line was consistently located between the external limiting membrane (ELM) and the ellipsoid zone on the intensity OCT image, positioned at 80.0% of the distance from the retinal pigment epithelium toward ELM at the fovea, 86.4% at 1 mm nasal, and 85.8% at 1 mm temporal. These findings suggest that the DCL corresponds to structural components within the photoreceptor inner segments, providing insights into photoreceptor structural organization.

## Introduction

Optical Coherence Tomography (OCT) is a widely used imaging technique that has become an essential tool in ophthalmology. OCT measures the time delay and intensity of backscattered light from different tissue layers based on the principles of optical low-coherence interferometry or optical frequency-domain interferometry^1,2^. This enables the generation of high-resolution cross-sectional images of microstructures in biological tissues, including the retina, optic nerve, and cornea. While conventional intensity-based OCT has transformed ophthalmic imaging and diagnostics, it primarily relies on the intensity of backscattered light, capturing only one aspect of tissue optical properties and leaving other contrast mechanisms underutilized.

Polarization-sensitive optical coherence tomography (PS-OCT) extends conventional OCT by providing both backscattering intensity and polarization-sensitive contrast^3–5^. By detecting changes in the polarization state of light, PS-OCT enables visualization of tissue properties that influence polarization. One such property is depolarization, in which the polarization state becomes spatially randomized. Tissues containing melanin pigments are known to exhibit depolarization properties^6,7^, making PS-OCT particularly suitable for observing melanin-rich structures such as the retinal pigment epithelium (RPE) and choroid^8–10^. While depolarization contrast has been demonstrated in pathological conditions, such as migrating RPE cells and hyperreflective foci in diseased eyes^11,12^, its application in the normal neural retina has received little attention, with only a single recent report by Ahmed et al.^13^.

Ahmed et al. analyzed parallel- and cross-polarization components of OCT signals but did not directly assess depolarization metrics, limiting the interpretability of their results. Additionally, their system employed full-field OCT, which is prone to multiple scattering and spatially coherent optical crosstalk due to the lack of confocal gating—a limitation not present in the flying spot scanning approach used in conventional OCT systems^14,15^. Therefore, further investigation is required to characterize the polarization properties of the normal neural retina using a scanning-based PS-OCT system and a direct depolarization metric.

In this study, we used PS-OCT to examine the outer retina and investigate the presence of consistent depolarization signals in healthy human eyes. Our PS-OCT system employs a flying-spot scanning approach, which minimizes coherent crosstalk and quantifies depolarization using polarimetric entropy, a metric effective for detecting depolarizing materials across a range of concentrations and independent of the incident polarization state^7,16^. We identified a moderately depolarizing layer, which we refer to as the depolarization contrast line (DCL), located between the external limiting membrane (ELM) and the ellipsoid zone (EZ). We statistically analyzed the visibility, age dependence, and regional variation of the DCL and discussed its potential structural and functional implications.

### Methods

### Participants

Between November 2023 and June 2024, 28 participants (56 eyes) without fundus disease were enrolled from Kobe City Eye Hospital. Participants were required to have no history of fundus diseases and no detectable fundus abnormalities based on slit-lamp microscopy and fundus photography. The cohort included 6 males and 22 females, aged 9–83 years (mean age: 57.1 ± 23.5 years; median: 65.5 years). All participants were phakic. The study was conducted in accordance with the Declaration of Helsinki and was approved by the Medical Ethics Committee of Kobe City Medical Center General Hospital (IRB No. 11000663). Informed consent was obtained from all participants before their inclusion in the study. For participants aged < 18 years, consent was obtained from the participants themselves, and additional consent was obtained from their legal guardians. Participants who did not provide informed consent were excluded. Since this study does not include identifiable personal data, specific consent for publication was not required.

### Image acquisition and image processing

Using a prototype PS-OCT device (Tomey Corporation, Nagoya, Japan), which was same as the commercially available model that received pharmaceutical approval in Japan, 100 repeated B-scan images were acquired over a 12-mm lateral scanning range, with 1,024 A-scans in both the horizontal and vertical directions through the fovea. The system was equipped with a frequency-swept laser at 1 μm wavelength with a repetition rate of 100 kHz, a fiber-optic interferometer with polarization sensitivity, and had optical resolutions of 8 μm and 30 μm in axial and lateral directions, respectively. The digital sampling size in the axial depth was 4.268 μm/pix in tissue. For polarimetric entropy signal processing, B-scan averaging was performed using ensemble averaging of a 4 × 4 covariance matrix, which was derived from a 2 × 2 Jones matrix, rather than directly averaging the polarimetric entropy B-scan images. The ensemble averaging was performed with a spatial kernel size of 5 × 11 pixels (axial × lateral, 21 μm × 129 μm) in addition to the temporal 100 pixels by the repeated B-scans. This approach improved the estimation quality of the polarimetric entropy in a similar way to that previously demonstrated for Stokes parameters by Götzinger et al.^17^ The spatial kernel size was same as the case of a single B-scan to prioritize denoising rather than spatial resolution. The remaining signal processing steps followed previously described methods^7,16^. In brief, the polarimetric entropy was defined mathematically according to von Neumann’s definition, which was known well in the fields of quantum mechanics, quantum information theory, and airborne radar polarimetry. The definition causally requested the range from 0 to 1 because of the logarithmic operation, which implied that the randomness of the multivariate signals could not be infinite but could be expressed with a certain upper limit. Images of the polarimetric entropy were shown with a pseudo-color map of Ametrine, which was designed to be readable for readers not only with normal color perception but also with color perception deficiencies^18^.

**Fig. 1:**
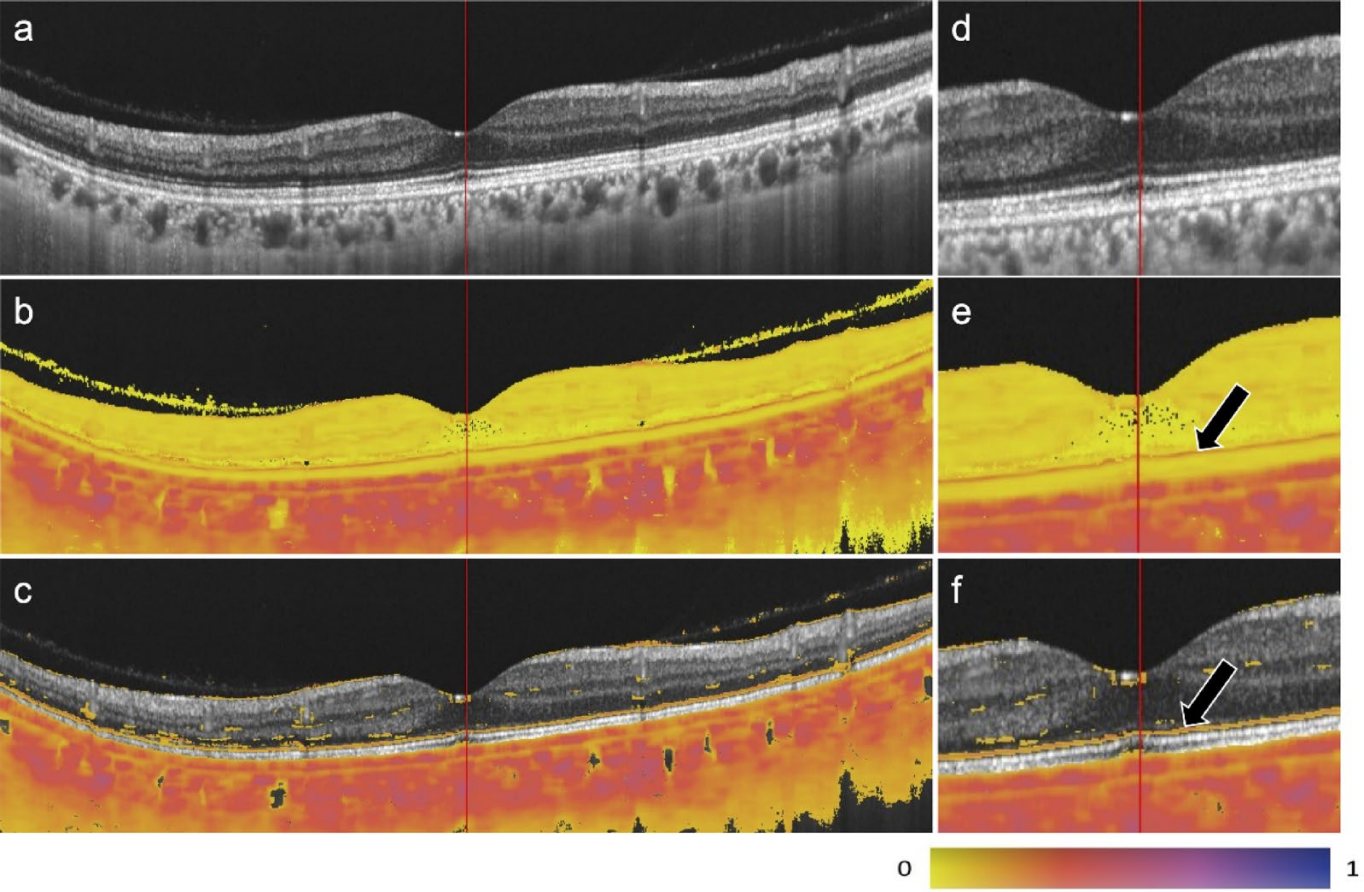
Representative case of a 42-year-old female. Images of (**a**) OCT intensity, (**b**) polarimetric entropy, and (**c**) polarimetric entropy superposed on OCT intensity for entropy values ≥ 0.1 are shown. Images (**d**–**f**) show magnified views of (**a**–**c**), respectively. A high-entropy response is observed in the outer retina (black arrow).

Figure 1 presents representative B-scan images. In addition to the RPE and choroid, which contain melanin pigments, a high-entropy response was detected in the outer retina, as indicated by the black arrows. Based on its consistent appearance and characteristics, we refer to this feature as “depolarization contrast line (DCL)”.

### Analysis of entropy values

**Fig. 2:**
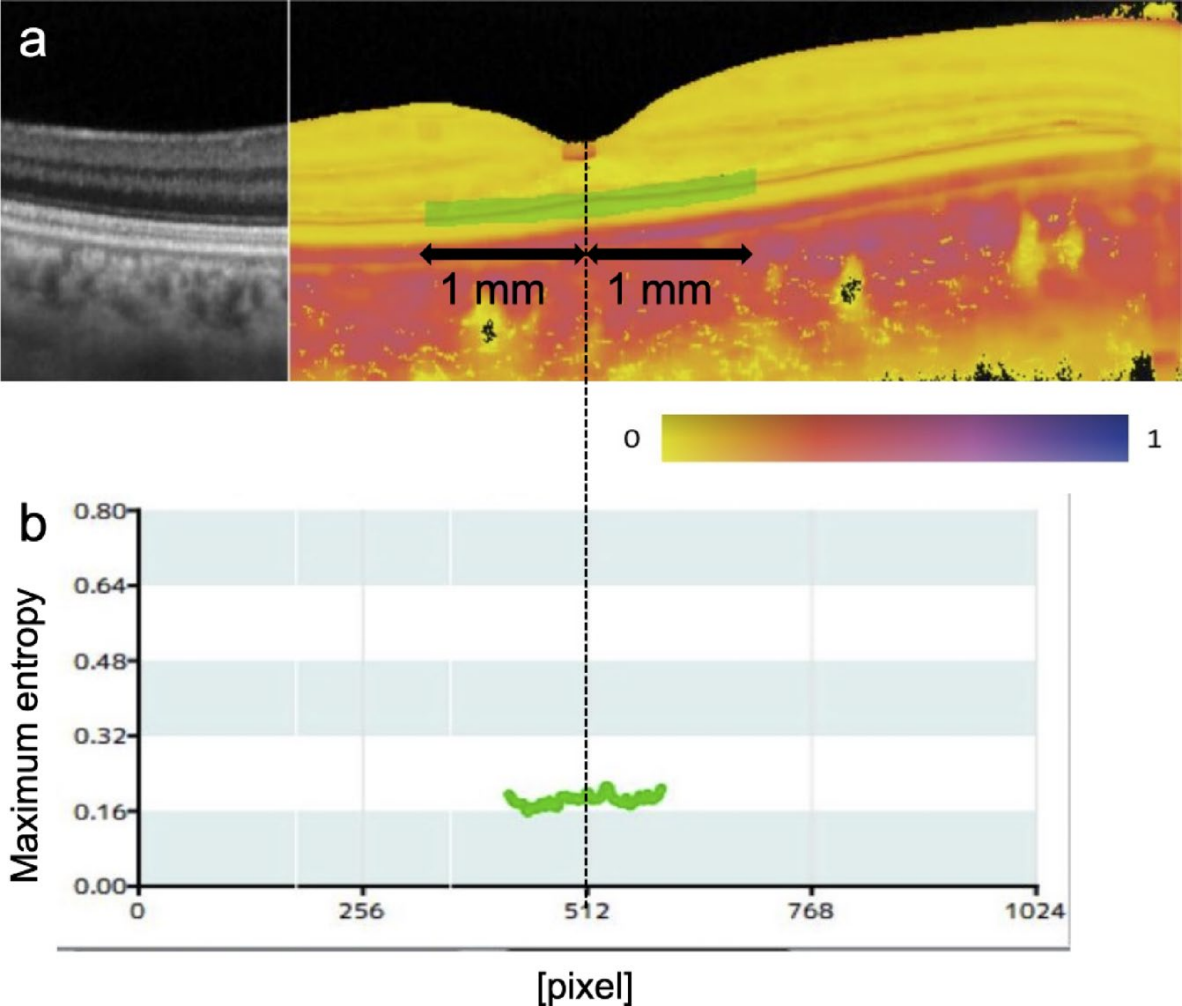
Method for entropy value analysis. (**a**) The analysis region was defined to be a slab with a thickness of 5 pixels (21 μm in tissue) centered at the DCL and extended laterally from the fovea to both temporal and nasal directions for 1 mm along the straight lateral direction in the B-scan image. (**b**) A graph displaying the maximum entropy for each A-scan within the analysis region. The position at 512 pixels corresponds to the fovea. The mean maximum entropy within the analysis region was calculated

To compute entropy values of the DCL and examine its reproducibility and correlation with age, regions of interest with a depth of 5 pixels were defined for each image to include the DCL while excluding the RPE. The maximum entropy for each A-scan within the analysis region was calculated, and the mean entropy value was determined within a 1-mm lateral range centered on the fovea (Fig. 2). Correlations between entropy values from horizontal and vertical scans were analyzed in cases where good-quality images were obtained. Participants with good-quality images in both eyes were selected to assess correlations between right and left eyes, using the average DCL entropy values from both horizontal and vertical B-scan images. Additionally, cases with at least one good-quality eye image were identified, and the correlation between age and entropy value (calculated as the average of the entropy values from the horizontal and vertical B-scan images) was analyzed for the eye with the higher entropy value, as some of the images obtained in this study were of poor quality due to factors such as cataracts that seem to reduce entropy.

### Image quality assessment

**Fig. 3:**
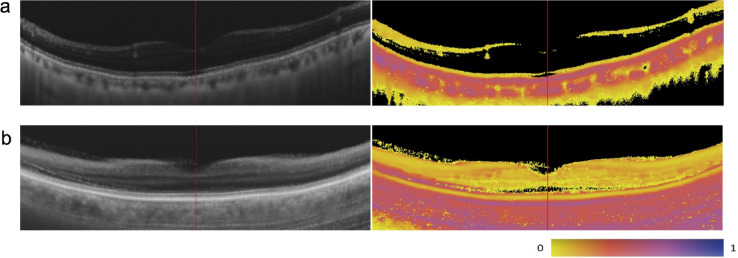
Examples of excluded cases in the entropy value analysis (in left images, intensity images; in right, entropy images). (**a**) A case with poor image quality due to cataracts (83-year-old male). The ELM is not continuously identified in the intensity image. (**b**) A case with motion artifacts (77-year-old female). Retinal boundaries and layer structures appear unclear in the intensity image.

For case selection, poor-quality images due to cataracts or motion artifacts were excluded. Image quality was assessed by visually inspecting intensity images, and poor-quality images were defined as those in which the ELM was not continuously identified or retinal boundaries and layer structures appeared unclear, as illustrated in Fig. 3.

### Analysis of the positional relationship between the DCL and retinal structures

**Fig. 4:**
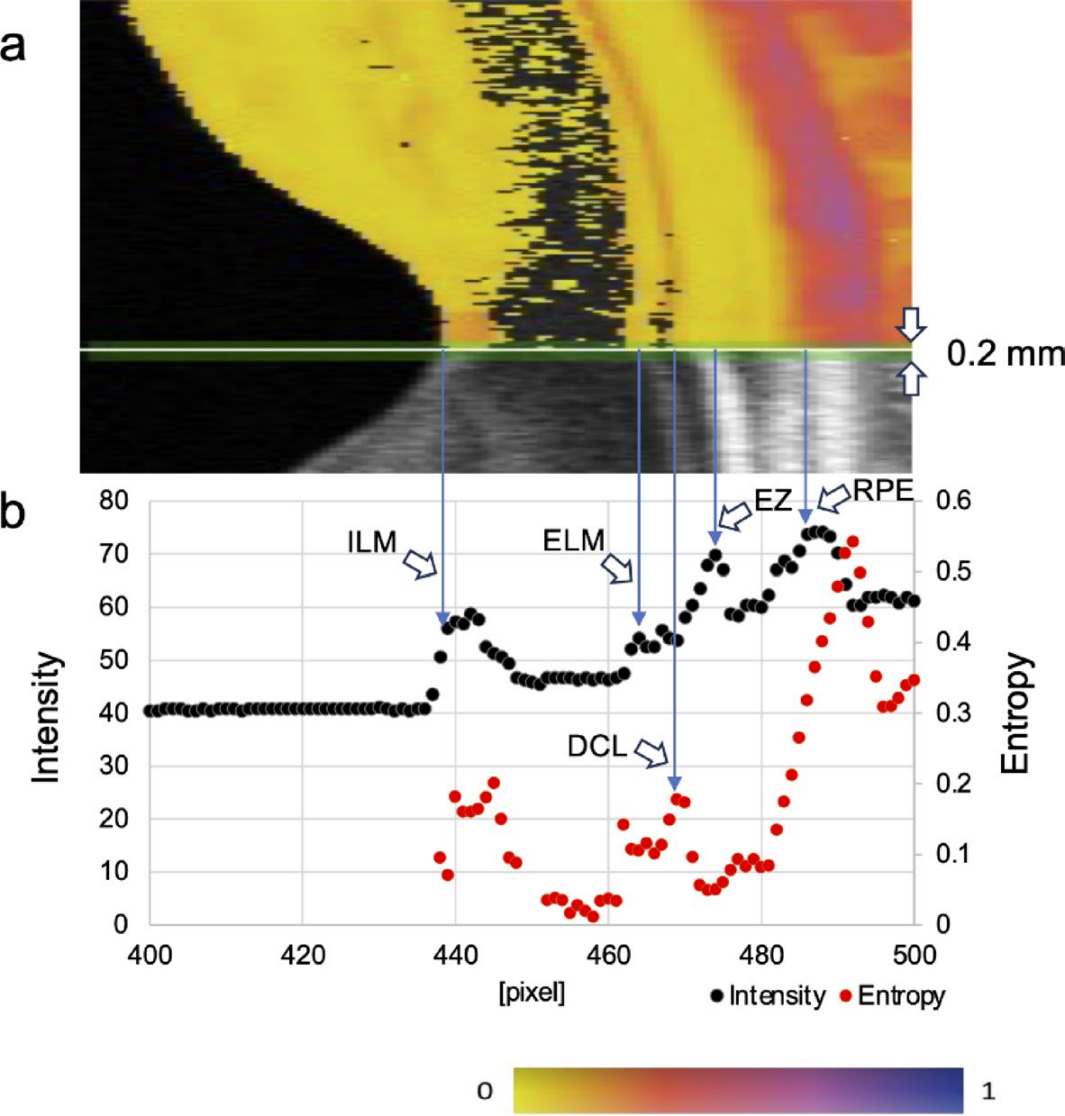
Methods for analyzing the positional relationship between DCL and retinal structures. (**a**) A 0.2 mm-wide region was defined using a horizontal scan. (**b**) Intensity and entropy values within the analysis region were plotted along the depth direction. Black represents intensity values, whereas red represents entropy values. The positions of the ELM, ellipsoid zone (EZ), RPE, and DCL were identified based on the peaks of these values.

The regional dependence of the DCL near the fovea was evaluated by comparing entropy and intensity images from 11 consecutive left-eye cases in which both the intensity image and the DCL were clearly visualized. The intensity and entropy values along the vertical line at the fovea and 1 mm nasal and temporal to the fovea were plotted. Peaks corresponding to the ELM, EZ, and RPE in the intensity image were identified, and the DCL peak in the entropy image was determined at a location between the ELM and RPE (Fig. 4). To standardize measurements, the thickness from the ELM to the RPE was normalized, and the relative depth positions of each peak were calculated by assigning 100% to the ELM and 0% to the RPE.

### Analysis of regional differences in DCL entropy values between the fovea and parafoveal regions

To investigate regional differences in entropy values, the entropy at the DCL was measured at three locations in these 11 eyes: the fovea, and positions 1 mm nasal and 1 mm temporal to the fovea. The entropy values at these three points were then compared.

### Statistical analysis

Spearman’s rank correlation coefficient was used to analyze entropy values, specifically examining the correlations (i) between vertical and horizontal entropy values, (ii) between the left and right eyes, and (iii) between entropy values and age. Friedman test was used to compare the DCL entropy values at the fovea and at 1 mm nasal and temporal to the fovea. Results were considered significant for *p* < 0.05. These statistical analyses were conducted using the JMP statistical software (version 17.0.0; JMP Statistical Discovery LLC, Cary, NC, USA).

## Results

**Fig. 5:**
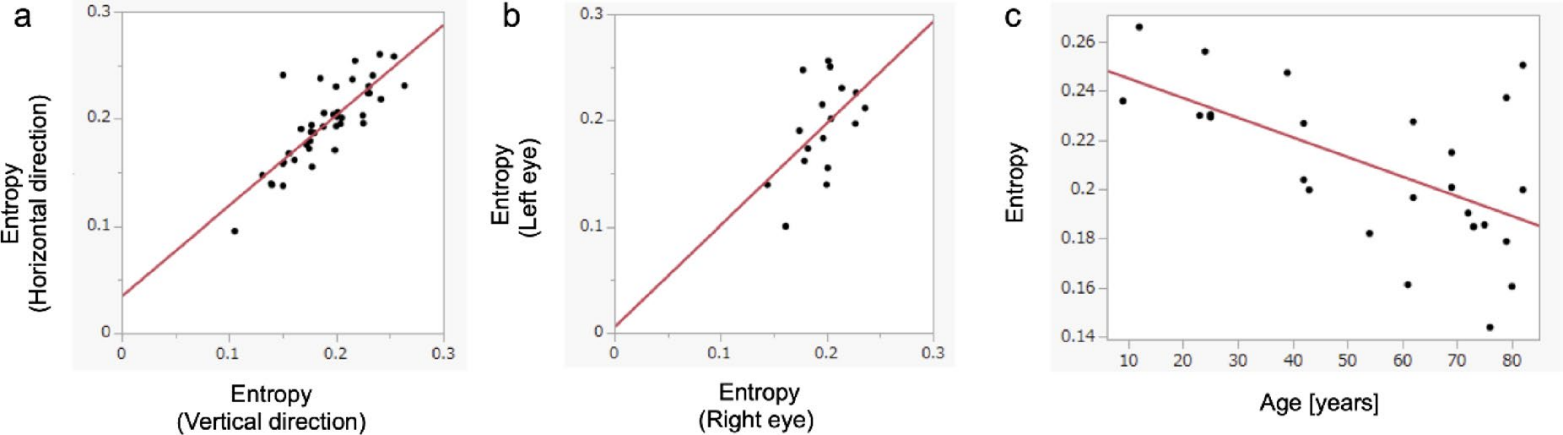
Scatter plot analysis of correlations. (**a**) Correlation of entropy values at the DCL between vertical and horizontal scans in cases where good-quality images were obtained (25 participants, 42 eyes), demonstrating a positive correlation (R² = 0.667, *p* < 0.0001). (**b**) Correlation of entropy values at the DCL between right and left eyes of participants with high-quality images in both eyes (17 participants, 34 eyes), demonstrating a positive correlation (R² = 0.240, *p* = 0.046). (c=**c**) correlation between entropy values and age of participants with at least one high-quality image (25 participants, 25 eyes), demonstrating a negative correlation (R² = 0.217, *p* = 0.019).

In the present study, the DCL was consistently observed as a continuous line without any image processing in cases where we could obtain fair intensity images. The mean entropy value in the region of interest containing the DCL, averaged across vertical and horizontal scans, was 0.191 ± 0.043 in all 56 eyes. The average entropy value was 0.193 ± 0.046 in the horizontal scans and 0.189 ± 0.046 in the vertical scans. Some cases exhibited unclear results due to poor image quality, which was attributed to cataracts or motion artifacts. Out of 56 eyes, good-quality images were successfully acquired in 42 eyes of 25 participants. When stratified by age group, the proportion of eyes with good-quality images was 40% in patients in their 80s, 64% in their 70s, 100% in those in their 30s to 60s, and 75% in patients in their 20s or younger. In the 42 eyes with good-quality images, a strong positive correlation was found between the entropy values of vertical and horizontal scan directions (R² = 0.667, *p* < 0.0001). Good-quality images were obtained for both eyes in 17 participants (34 eyes), with exhibiting a positive correlation between the entropy values of the right and left eyes (R² = 0.240, *p* = 0.046). At least one eye with good-quality imaging was obtained in 25 participants. Among these, a negative correlation was observed between entropy and age in 25 eyes with higher entropy values (R² = 0.217, *p* = 0.019) (Fig. 5).

**Fig. 6:**
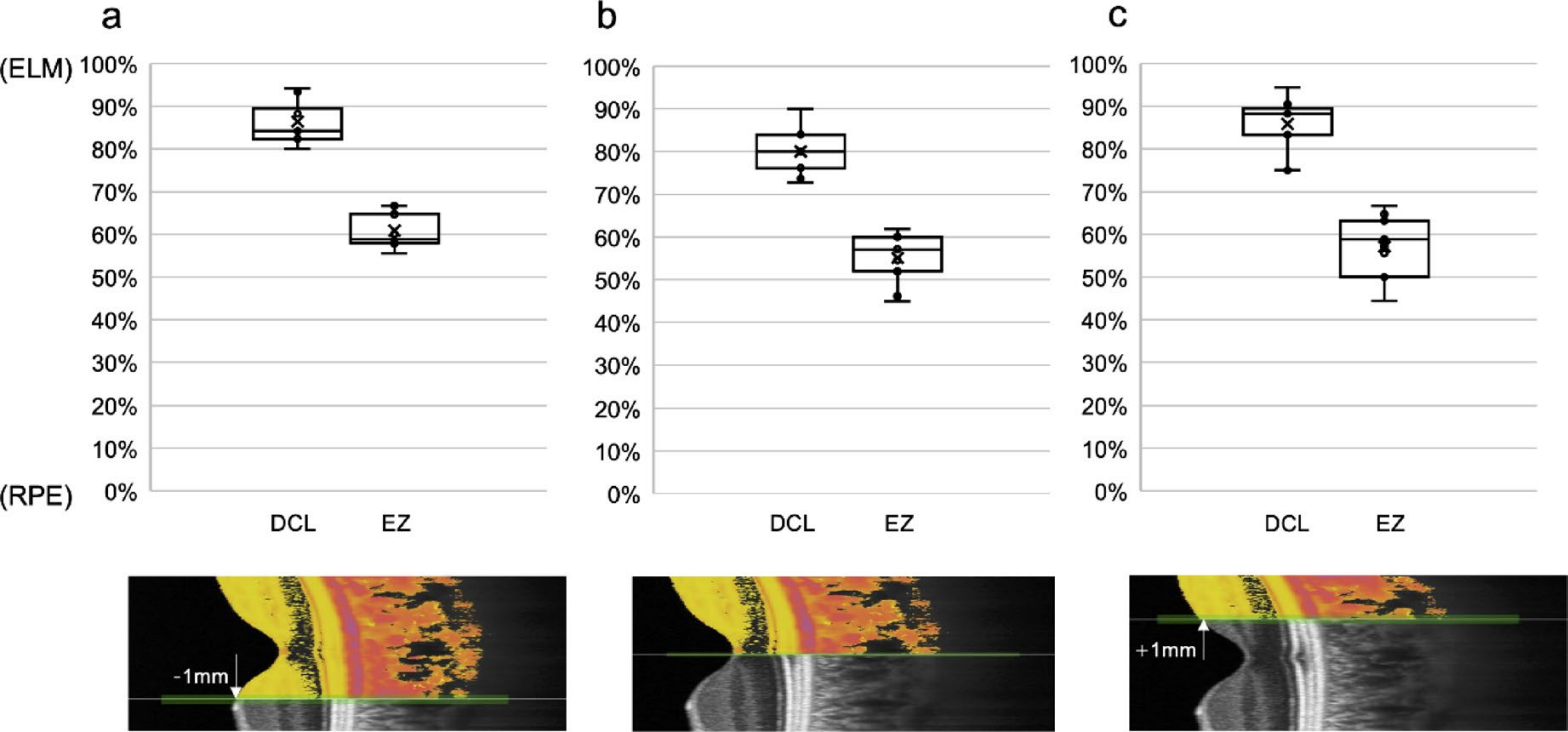
Box plot showing the relative positions of the DCL and the EZ, with the ELM set at 100% and the RPE at 0% (*n* = 11). (**a**) At 1 mm nasal to the fovea, the DCL was located at 86.4 ± 4.7%, and the EZ at 60.9 ± 4.0%. (**b**) At the fovea, the DCL was located at 80.0 ± 5.0%, and the EZ at 55.1 ± 5.5%. (**c**) At 1 mm temporal to the fovea, the DCL was located at 85.8 ± 6.2%, and the EZ (IS/OS) at 57.1 ± 6.8%.

**Fig. 7:**
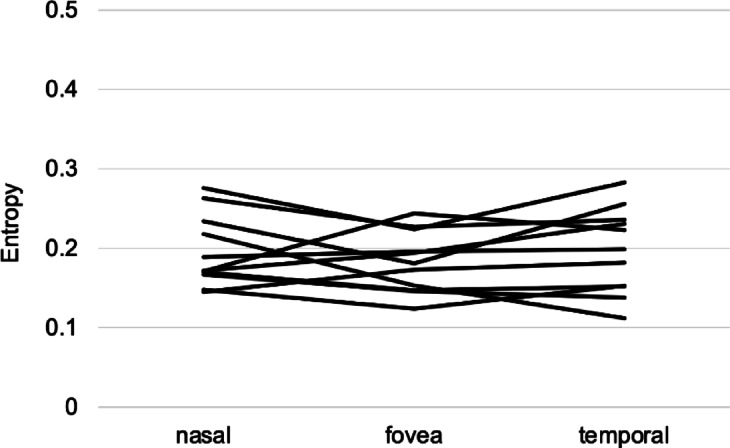
A line plot showing the DCL entropy values at the fovea and parafovea. No significant differences were observed in the DCL entropy values between the fovea and the positions 1 mm nasal and temporal to the fovea (*p* = 0.2335).

Finally, the depth positions of the DCL relative to the ELM and EZ lines were analyzed in the outer retinal structure of 11 eyes (11 participants) where DCL was clearly observed. At 1 mm nasal to the fovea, with 100% assigned to the ELM and 0% to the RPE, the DCL was located at 86.4 ± 4.7%, and the EZ at 60.9 ± 4.0% (Fig. 6a). At the fovea, the DCL was located at 80.0 ± 5.0%, and the EZ at 55.1 ± 5.5% (Fig. 6b). At 1 mm temporal to the fovea, the DCL was located at 85.8 ± 6.2%, and the EZ at 57.1 ± 6.8% (Fig. 6c). The DCL was consistently located between the ELM and EZ across all lateral positions (Fig. 6). Additionally, comparison of DCL entropy values across the three locations revealed no statistically significant variation (*P* = 0.2335) (Fig. 7), suggesting that the DCL represents a potentially continuous and consistent structure across the fovea.

## Discussion

In this study, the DCL was consistently observed in the outer retina of normal eyes using PS-OCT. Entropy analysis demonstrated a significant positive correlation between the vertical and horizontal scan directions, as well as between the left and right eyes, confirming its reproducibility. Additionally, a comparison with intensity images revealed that the HE line was consistently located between the ELM and the EZ.

In principle, low signal-to-noise ratio (SNR) narrows the dynamic range of the polarimetric entropy. As shown in Fig. 5 of Yamanari et al. (2016)^16^ previously, the SNR of a few dB ensures a dynamic range from 0 to 0.2 in the polarimetric entropy. The SNR between the ELM and the EZ was more than 10 dB as shown in Fig. 4 of our manuscript, ensuring the dynamic range from 0 to more than 0.6 in the polarimetric entropy.

Conventional OCT imaging of the outer retina typically reveals four distinct hyper-reflective bands. It is generally accepted that the first band corresponds to the ELM, whereas the fourth band represents the retinal pigment epithelium (RPE)/Bruch’s membrane complex^19,20^. In contrast, the precise anatomical correlations of the second and third bands remain controversial^21^.

Historically, the second band has been considered the inner segment/outer segment (IS/OS) junction. However, a comparison between an anatomically derived scale model of the outer retinal morphology and OCT imaging suggested that the second band corresponds to the mitochondria-rich inner segment ellipsoid, whereas the third band likely originates from the interdigitation zone^19^. Accordingly, in 2014, the International OCT Nomenclature Meeting designated the second band as the “Ellipsoid zone of the photoreceptors” and the third band as “Cone interdigitation with RPE.”^20^ However, studies utilizing adaptive optics OCT (AO-OCT), which provides improved lateral resolution, have proposed alternative interpretations. The second band appears too thin and is positioned too far from the ELM to represent the EZ, suggesting that the second and third bands more likely correspond to the IS/OS junction and photoreceptor OS tips, respectively^22,23^. Notably, differences in device characteristics and the retinal imaging area may contribute to this discrepancy between conventional OCT and AO-OCT^21^. Furthermore, studies employing ultra-high-resolution visible-light OCT have demonstrated that the second band splits into two distinct bands in the peripheral retina^24^. This observation has been attributed to differences in IS length between cones and rods, further supporting the hypothesis that the second band originates from the IS/OS junction. In contrast, cell-specific immunostaining studies comparing human retinal tissues with OCT images have proposed that the second band corresponds to the EZ, and the third band aligns with the phagosome zone^25^.

The precise anatomical origins of the second and third bands remain unclear. Irrespective of the specific anatomical interpretation, the DCL observed between the ELM and EZ, or the second band in this study, likely corresponds to a structural component within the photoreceptor inner segment.

Ahmed et al. analyzed signals from both parallel and cross-polarization in the outer retina using polarization-sensitive full-field swept-source OCT^13^. Regarding the second band, they proposed that parallel polarization captures reflections at the IS/OS boundary, whereas cross polarization detects multiple scattering from mitochondria within the EZ, suggesting that the second band may contain signals from both the EZ and IS/OS. Their study also identified a region of relatively high cross-polarization intensity immediately below the high-intensity ELM line. However, as depolarization was not quantitatively assessed, whether the cross-polarization findings stemmed from depolarization effects remains unclear. Nevertheless, their observations may reflect a phenomenon similar to that identified in the present study.

Melanin is a well-known depolarizing substance in ophthalmology^6,7^, whereas PS-OCT studies of coronary arteries have reported depolarization in adipose tissue and macrophages^26^. In the photoreceptor inner segment, lipofuscin was previously identified in the eyes of humans older than 30 years^27^, likely accumulating due to reduced clearance of damaged inner segment organelles^28^. Given the optical refractive index distribution of lipofuscin, which is similar to that of adipose tissue, it can be hypothesized that it is a potential contributor to the DCL. However, in the present study, the DCL was observed even in young individuals aged 12 years or younger, and entropy values showed a negative correlation with age. These findings contradict the lipofuscin hypothesis and may instead reflect other age-related changes.

Nevertheless, as this study focused on phakic eyes, the potential influence of entropy reduction due to cataracts, which may theoretically limit the available dynamic range of entropy, cannot be entirely excluded. Further investigations using high-quality imaging are warranted to clarify the potential impact of lens opacities on entropy measurements.

Another possibility that the DCL is an artifact should be carefully considered. We can categorize two possible sources of the artifacts, systematic errors and artifacts originating from tissue structure. As for the systematic errors, it has been known that polarization mode dispersion (PMD) in an optical interferometer causes the artifacts in PS-OCT^29^. Several PS-OCT systems corrected the PMD numerically^30–32^. In our PS-OCT system used in this study, the PMD is superposed on the other systematic signal distortions caused by the parallel detection scheme, and these are numerically corrected in the signal processing^33^. Even if the PMD were not corrected sufficiently, it degraded almost all regions of the PS-OCT images without localizing in the specific tissue region. Therefore, the possibility of the artifact caused by the systematic errors is denied. As for the artifacts originating from tissue structure, we cannot exclude the possibility of the artifact at the tissue boundaries between retinal layers, because the spatial kernel to calculate the polarimetric entropy at the tissue boundaries includes polarization properties of the multiple tissues. However, this hypothesis contradicts our experimental findings, where neither the ELM nor the EZ showed depolarization. In summary, we do not have any remaining hypothesis that the DCL is the artifact, and hence, we believe that our finding reflects real polarimetric property in the inner segment.

We note that additional lines with weak polarimetric entropy were visible at the inner limiting membrane on the neural retina, near the anterior and posterior ends of the inner nuclear layer, and near the posterior end of the outer nuclear layer that was anterior to the ELM as shown in Figs. 1c and 2a. Although we excluded these regions from the analysis in this study because of their subtle appearances, we also do not have any hypothesis that supports the possibility of artifacts for these appearances. It may be intriguing to investigate them in future.

This study has several additional limitations. The sample size was relatively small, and while findings were consistent, larger studies are needed to confirm their generalizability. Image quality varied, with some scans excluded due to cataracts and motion artifacts. High-resolution imaging approaches may improve reproducibility and reduce exclusion rates. The anatomical basis of the DCL remains unconfirmed by histological validation such as direct comparisons with retinal tissue samples. Although we used the same spatial kernel size of the ensemble averaging to calculate the polarimetric entropy as the case of a single B-scan, we may be able to shrink the size to improve the spatial resolution of the polarimetric entropy while considering the balance between the denoising and the spatial resolution. It is our future issue to take the best balance by the tuning of processing parameters.

The EZ or second band in the outer retina is frequently utilized as a biomarker of visual function in ophthalmic imaging^34–37^. If the DCL represents a distinct substructure within the inner segment, it may provide a new biomarker for photoreceptor integrity. Understanding its role could enhance the diagnostic capabilities of PS-OCT, particularly in detecting early degenerative changes. Future research should focus on longitudinal studies to track DCL changes over time and in relation to retinal disease. Investigating its presence in conditions such as age-related macular degeneration or inherited retinal dystrophies could clarify whether it serves as a marker of structural or functional decline. High-resolution imaging techniques, such as adaptive optics OCT and visible-light OCT, could help resolve its fine structure and confirm its anatomical origin. Functional studies correlating entropy values with visual acuity, electrophysiological responses, or metabolic activity may further establish its clinical relevance.

## Data Availability

The data supporting the findings of this study are available from the corresponding author, M. M., upon reasonable request.
